# Genetic diversity and phylogenetic characteristics of human adenovirus strains 40/41 circulating in Yantai, China, during 2017–2019

**DOI:** 10.1128/aem.00983-25

**Published:** 2025-11-25

**Authors:** Peihua Niu, Pengcheng Du, Zhenlu Sun, Xiuhui Yao, Yiming Zhao, Ping Cheng, Qun Yang, Zetang Zhang, Xuejun Ma, Ji Wang

**Affiliations:** 1 National Key Laboratory of Intelligent Tracking and Forecasting for Infectious Diseases, NHC Key Laboratory of Medical Virology and Viral Diseases, National Institute for Viral Disease Control and Prevention, Chinese Center for Disease Control and Prevention 12415, Beijing, China; 2 Medical Research Center, Beijing Institute of Respiratory Medicine and Beijing Chao-Yang Hospital, Capital Medical University 12517, Beijing, China; 3 Qitan Technology Ltd., Chengdu, China; 4 Yantai Center for Disease Control and Prevention 606828, Yantai, Shandong, China; University of Nebraska-Lincoln, Lincoln, Nebraska, USA

**Keywords:** human adenovirus, whole-genome sequencing, phylogenetics, recombination, structural, AlphaFold3

## Abstract

This study systematically characterizes the genetic diversity, phylogenetic relationships, recombination events, and structural variations of human adenovirus serotypes 40 and 41 (HAdV-F40/41) circulating in Shandong Province, China, between 2017 and 2019. A total of 2,221 stool samples were collected from patients presenting with acute gastroenteritis in Yantai. Enteric adenoviruses were identified using real-time quantitative PCR targeting the hexon gene. Whole-genome sequencing was performed via nanopore and Illumina platforms, enabling high-resolution phylogenetic reconstruction with IQ-TREE and the identification of recombination events using RDP5 and SimPlot. Evolutionary dynamics were inferred through Bayesian molecular clock analysis, while AlphaFold3-based structural modeling was used to assess the impact of genomic changes on the hexon protein. HAdV-F40/41 was detected in 94 samples (4.23%) with year-to-year variability in prevalence. Phylogenetic analysis revealed the co-circulation of multiple genetically distinct lineages, while recombination mapping identified key breakpoints, notably within the hexon and pVII genes. The estimated recent divergence of dominant lineages suggests ongoing adaptive evolution. Structural modeling of the recombinant strain SD376 identified 14 amino acid substitutions and three deletions relative to the reference strain OP378826.1, without substantial conformational alteration. These findings provide critical insights into the genomic plasticity and evolutionary potential of HAdV-F40/41, underscoring the importance of continuous genomic surveillance. The observed mutations and recombination patterns may facilitate immune escape and viral persistence, reinforcing the urgent need for improved monitoring strategies and the development of targeted vaccines.

**IMPORTANCE:**

Human adenovirus serotypes 40 and 41 (HAdV-F40/41) are among the leading viral causes of pediatric acute gastroenteritis worldwide, yet their genetic diversity and evolutionary dynamics remain poorly characterized in many regions, including China. In this study, we systematically investigated HAdV-F40/41 strains circulating in Yantai, Shandong Province, from 2017 to 2019. Using a combination of real-time PCR, nanopore sequencing, and next-generation sequencing, we elucidated the phylogenetic relationships, recombination events, and structural features of circulating strains. Our results reveal extensive genetic variability and the emergence of recombinant lineages with altered antigenic profiles, underscoring the role of adaptive evolution and potential immune escape. The identification of key mutations and recombination hotspots in hexon and pVII genes provides important molecular markers for surveillance and risk assessment. These findings enhance our understanding of HAdV-F40/41 evolution and highlight the urgent need for continuous genomic monitoring and targeted vaccine development to mitigate the public health burden of adenoviral gastroenteritis.

## INTRODUCTION

Human adenovirus serotypes 40 and 41 (HAdV-F40/41) represent major viral pathogens responsible for pediatric acute gastroenteritis (AGE) globally, with a particularly pronounced impact on children under the age of 5 (1). In the wake of widespread rotavirus vaccination, HAdV-F40/41 has emerged as a predominant cause of viral diarrhea, with profound clinical and public health ramifications (2). Although several studies have reported the detection and partial genetic characterization of HAdV-F40/41 strains, most have focused on limited genomic regions, such as the hexon (3, 4) or fiber gene (5), without comprehensive whole-genome analysis. Little is known about the full spectrum of recombination events, genome-wide mutation patterns, or the integration of regional and global phylogenetic data (6). Moreover, prior studies have rarely linked molecular findings with structural modeling or clinical/epidemiological context (7). Despite several surveillance studies, investigations into the regional genetic diversity of HAdV-F40/41 in China remain limited and largely confined to a few selected areas. Comprehensive phylogenetic analyses of strains circulating in coastal eastern cities such as Yantai are particularly scarce, leaving significant gaps in our understanding of the local evolutionary dynamics of these viruses (8).

In this study, we address these gaps by performing whole-genome sequencing, detailed recombination mapping, global phylogenetic analysis, and AlphaFold3-based protein structural predictions for HAdV-F40/41 strains circulating in Yantai, China. This integrated approach allows us to provide new insights into the evolution, diversity, and potential functional implications of circulating enteric adenoviruses.

## RESULTS

### Study population characteristics

Between January 2017 and December 2019, a total of 2,221 stool samples were collected from patients diagnosed with AGE in Yantai, Shandong Province, China. The number of samples collected each year was 713 in 2017, 779 in 2018, and 729 in 2019.

Real-time PCR detection identified 94 adenovirus-positive cases, resulting in an overall positivity rate of 4.23% (94/2,221). The annual positivity rates were 3.51% (25/713) in 2017, 6.55% (51/779) in 2018, and 2.47% (18/729) in 2019, indicating notable fluctuations in HAdV detection rates across the study period. Among the 94 adenovirus-positive samples, 37 were successfully sequenced and assigned to specific subtypes. Of these, four cases were classified as HAdV-F40, and 33 cases were identified as HAdV-F41, underscoring the predominance of HAdV-F41 among enteric adenovirus infections in this cohort. The Ct values for positive cases ranged from 10.47 to 37.26, with a median of 29.54, indicating variable viral loads among the patients. The age distribution of adenovirus-positive cases ranged from 0.17 to 73 years, with a median age of 2 years, highlighting a higher prevalence in younger individuals. The gender distribution was balanced, with 17 male cases (45.9%) and 16 female cases (43.2%), suggesting no significant sex-based difference in infection rates. By integrating nanopore sequencing results with next-generation sequencing (NGS) data, with a sequencing depth of ≥50× and genome coverage exceeding 80%, 33 full-length adenoviral genomes were successfully obtained, providing a robust foundation for subsequent sequence-based analyses.

### SNV Analysis

To characterize the genetic variability of HAdV-F40 and HAdV-F41, SNVs were identified through comparative analysis against subtype-specific reference genomes.

**Fig 1:**
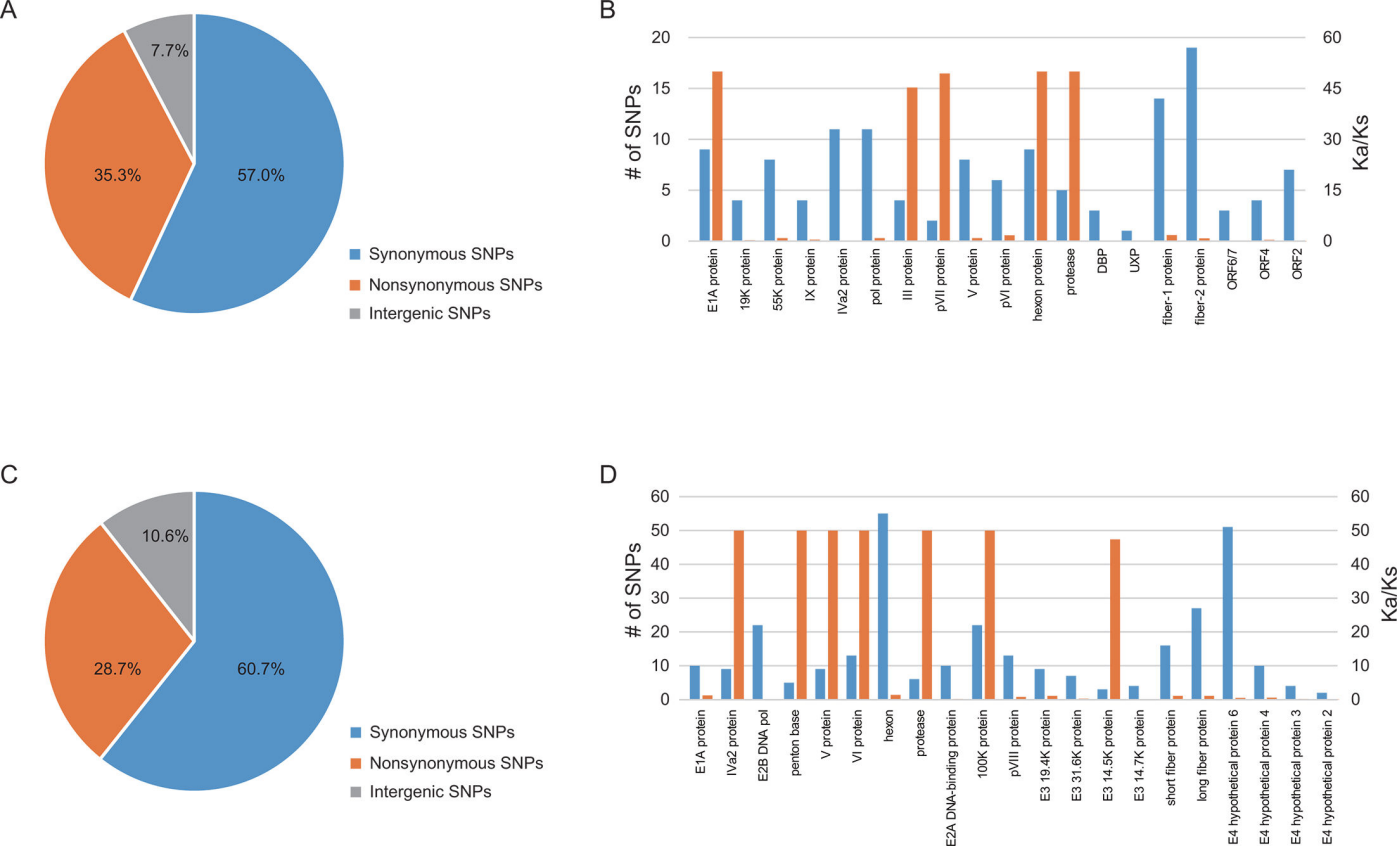
SNPs identified in the sequenced genomes of the two subtypes. The total number of SNPs identified in the sequenced genomes of HAdV-F40 (**A**) and HAdV-F41 (**C**). The number of SNPs and the Ka/Ks ratio for each gene of HAdV-F40 (**B**) and HAdV-F41 (**D**).

For HAdV-F40, a total of 453 SNVs were detected across the four sequenced genomes, comprising 258 synonymous and 160 non-synonymous mutations. Genes encoding the long fiber protein (19 SNVs), short fiber protein (14), DNA polymerase (11), IVa2 (11), hexon (9), and E1A (9) proteins exhibited the highest frequency of mutations. Additionally, genes encoding hexon (Ka/Ks = 50.0), E1A (50.0), protease (50.0), pVII (49.4), and protein III (45.3) displayed elevated Ka/Ks ratios, suggesting the influence of selective pressures driving viral evolution (Fig. 1).

For HAdV-F41, 527 SNVs were identified across 30 sequenced genomes, including 320 synonymous and 151 non-synonymous variants. Excluding genes encoding hypothetical proteins, the most frequently mutated genes were those encoding hexon (55 SNVs), long fiber protein (27), E2B DNA polymerase (22), 100K protein (22), and short fiber protein (9). Genes encoding IVa2, penton base, V protein, VI protein, protease, and 100K protein exhibited elevated Ka/Ks ratios, indicating ongoing adaptive evolution within these genomic regions (Fig. 1).

These results highlight the considerable genetic heterogeneity within HAdV-F40 and HAdV-F41, revealing substantial sequence variability in key structural and functional genes. The observed mutation patterns, particularly within antigenic determinants and replication-associated proteins, may have significant implications for viral fitness, immune evasion, and host adaptation, underscoring the need for further investigation into their phenotypic consequences.

### Phylogenetic analysis of HAdV-F40/41 strains

**Fig 2:**
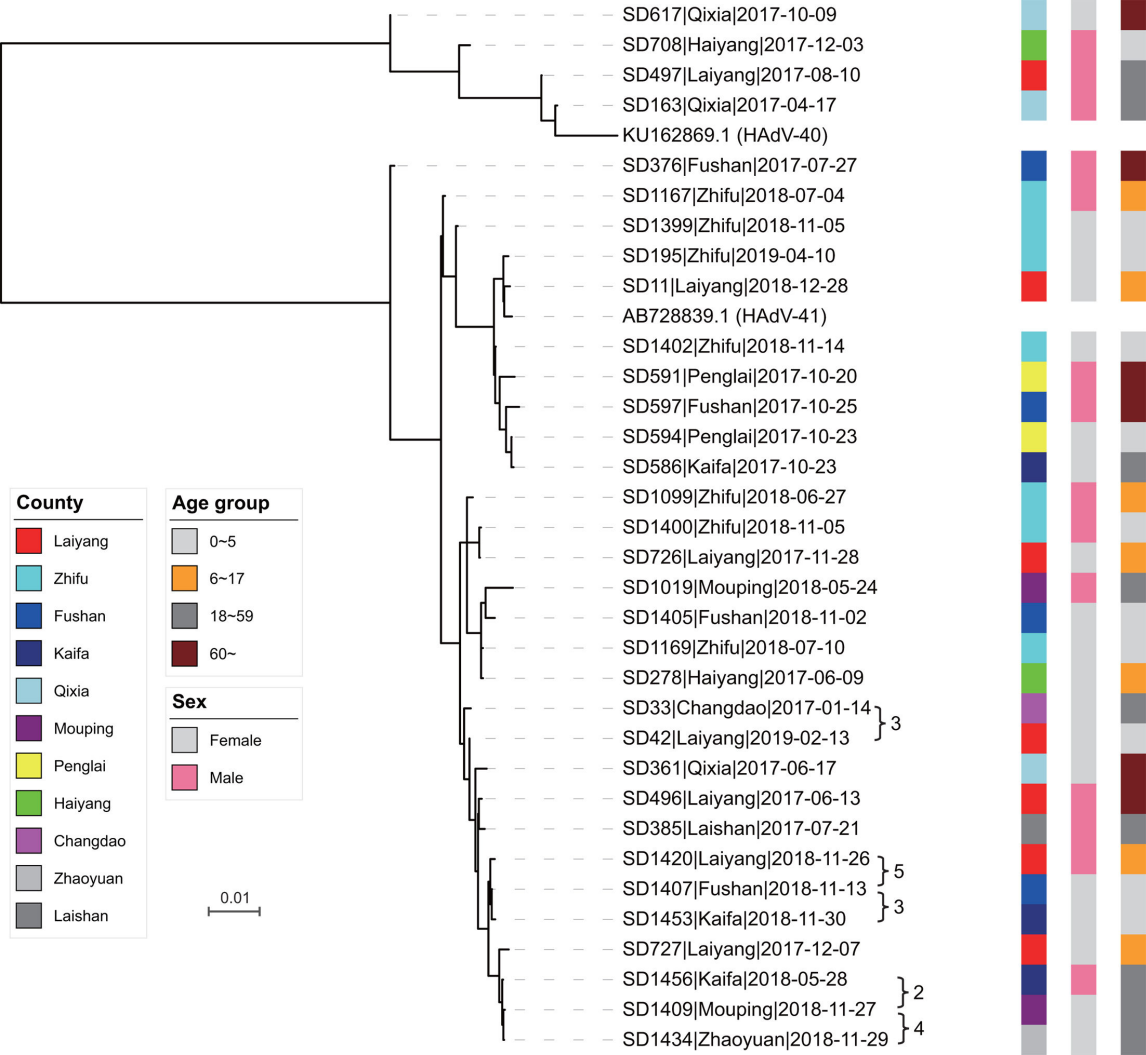
Phylogenetic tree of the sequenced genomes. The tree illustrates the phylogenetic relationships of the sequenced genomes. The collection locations, age group, and sex of the patients are annotated beside the tree.

To validate the subtyping results and assess the genetic diversity of the viral sequences, phylogenetic analysis was conducted using the maximum likelihood (ML) method based on the complete genome sequences of HAdV-F40 and HAdV-F41 (Fig. 2). The four HAdV-F40 genomes and 33 HAdV-F41 genomes clustered with their respective reference sequences, forming two distinct and well-supported phylogenetic clades corresponding to the two subtypes.

Despite belonging to the same subtype, HAdV-F40 genomes exhibited considerable genetic divergence, with 74-592 pairwise SNVs, reflecting their phylogenetic distances and suggesting that these strains were not derived from recent direct transmission events. In contrast, the 29 HAdV-F41 genomes displayed 2–344 pairwise SNVs, with two distinct clusters of three genomes each, characterized by fewer than five pairwise SNVs. These clusters exhibited a high degree of genetic similarity and were phylogenetically adjacent. Notably, all six genomes within these clusters were collected in 2018, despite originating from five different counties, suggesting the occurrence of localized transmission events during that period.

No clear clustering trends were observed in relation to patient gender, age group, or county of origin, indicating that the phylogenetic distribution of strains was not influenced by these demographic factors (Fig. 2). These findings underscore the genetic heterogeneity of circulating HAdV-F40 and HAdV-F41 strains and provide valuable insights into potential transmission dynamics within the study population.

### Evolutionary analysis of HAdV-F40/41 lineages

To explore the dissemination and evolutionary patterns of HAdV-F40 and HAdV-F41, a global phylogenetic analysis was conducted by integrating the genomes generated in this study with publicly available sequences from the NCBI GenBank database, representing diverse geographical regions.

For HAdV-F40, the analysis encompassed four sequences from this study along with 31 publicly available genomes, the majority of which (30/31) were sourced from Kenya, reflecting a geographically constrained distribution. In contrast, for HAdV-F41, 30 sequences from this study were analyzed in conjunction with 134 publicly available genomes, with the largest contributions from China (10), the United Kingdom (41), Germany (30), and Kenya (28), indicating a more widespread global distribution, particularly with notable representation from European and African regions. Bayesian evolutionary estimation was performed on 35 HAdV-F40 sequences and 164 HAdV-F41 sequences, incorporating strain collection dates to infer the temporal dynamics of their evolutionary trajectories.

**Fig 3:**
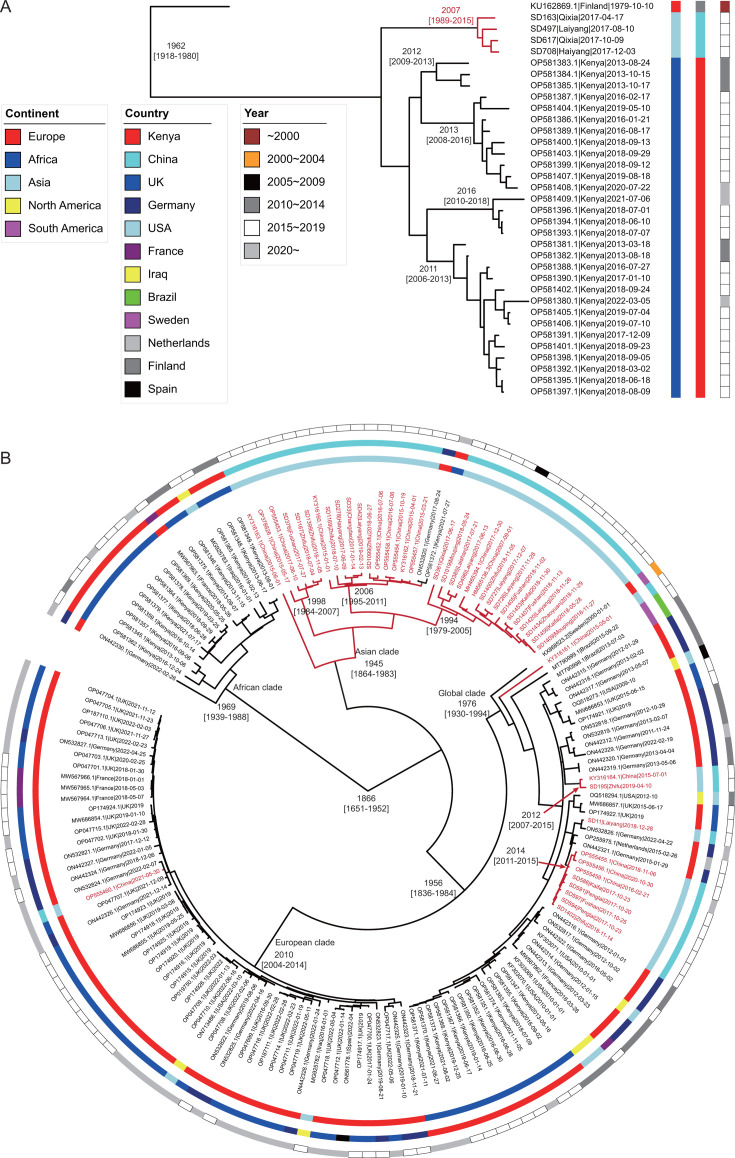
Bayesian evolutionary estimation results of the two subtypes, HAdV-F40 (**A**) and HAdV-F41 (**B**). The continents and countries of sample collection, along with the collection years, are annotated beside the tree. The branches corresponding to the genomes sequenced in this study are highlighted in red.

For HAdV-F40, the lognormal uncorrelated relaxed clock model estimated an evolutionary rate of 1.9 × 10⁻⁴ substitutions per site per year (95% highest posterior density [HPD] = 0.4–3.1 × 10⁻⁴), corresponding to an accumulation of approximately 6.5 single-nucleotide polymorphisms (SNPs) per genome per year (95% HPD = 1.4–10.6). The most recent common ancestor (MRCA) of HAdV-F40 was inferred to have emerged around 1962 (95% HPD = 1,918–1,980), with the sequence closest to this MRCA originating from Finland in 1979 (Fig. 3A). The phylogenetic structure revealed two major clades emerging in 1994: one comprising sequences from China (this study) and the other from Kenya. The Kenyan lineage exhibited further diversification, forming four subclades between 2011 and 2016, suggesting ongoing circulation and potential outbreaks, whereas the Chinese lineage remained evolutionarily distinct, indicating limited intercontinental transmission. For HAdV-F41, the estimated mean evolutionary rate was 5.4 × 10⁻⁵ substitutions per site per year (95% HPD = 2.2–8.1 × 10⁻⁵), corresponding to an accumulation of 1.8 SNPs per genome per year (95% HPD = 0.8-2.8). The MRCA of HAdV-F41 was inferred to have emerged around 1866 (95% HPD = 1,651–1,952, Fig. 3B), significantly earlier than that of HAdV-F40. Phylogenetic reconstruction revealed four major clades that originated from the MRCA in the mid-20th century, suggesting a long-term stable evolutionary trajectory with multiple diversification events.

These findings underscore the distinct evolutionary patterns and transmission dynamics between HAdV-F40 and HAdV-F41. HAdV-F40 exhibits a relatively recent emergence with regional clustering and limited geographic spread, whereas HAdV-F41 demonstrates long-term global circulation with sustained diversification, reflecting its broader transmission potential and evolutionary stability.

### Global dissemination and transmission of HAdV-F41

Mapping the global dissemination and evolutionary dynamics of HAdV-F41 revealed substantial genetic diversity, with distinct phylogenetic clusters structured by geographic origin. The global phylogenetic analysis identified four major clades, three of which exhibited strong regional clustering, predominantly composed of sequences from Africa (Kenya), Asia (China), and Europe.

Within these regional clades, 14 of 17 genomes (82.4%) in the African clade were sourced from Kenya, 33 of 35 genomes (94.3%) in the Asian clade were collected from China, and 53 of 56 genomes (94.6%) in the European clade were sampled from European countries. This pattern of localized viral evolution suggests that HAdV-F41 strains have adapted to specific populations and environmental conditions, with limited intercontinental transmission. The pronounced geographic segregation of these clusters implies that host distribution and ecological factors play a crucial role in shaping viral evolution. Nevertheless, sequences from other regions were also identified within these clades, indicating that sporadic cross-regional transmission events have occurred.

In contrast, the fourth clade, referred to as the “global clade,” exhibited a distinctly different transmission pattern, encompassing genomes from five continents: Europe (23/55, 41.8%), Africa (13/55, 23.6%), Asia (12/55, 21.8%), North America (5/55, 9.1%), and South America (2/55, 3.6%). The widespread geographic distribution of this clade suggests a historically extensive dissemination, facilitating intercontinental viral exchange.

Among the 46 HAdV-F41 genomes from China, 33 clustered within the Asia clade, one was placed in the European clade, and 12 were situated in the global clade. Notably, these 12 globally associated Chinese genomes were distributed across four distinct lineages, all demonstrating close genetic relationships with European strains, suggesting potential direct or indirect transmission events between Asia and Europe. Furthermore, two genetically distinct clusters within this clade were identified, comprising two and eight genomes, respectively, with estimated emergence dates in 2012 and 2014, indicating recent intercontinental transmission followed by local circulation.

These findings highlight the complexity of HAdV-F41 transmission dynamics, revealing both regionally confined evolution and long-distance viral spread. The structured nature of the three regional clades supports the hypothesis of localized adaptation, while the global clade underscores the role of international viral exchange in shaping genetic diversity. This further emphasizes the necessity for enhanced global surveillance to monitor emerging transmission pathways.

### Recombination in HAdV-F40/41

No significant intratypic recombination events were detected within the HAdV-F40 genomes. Phylogenetic analysis and sequence alignments indicated that the genetic diversity observed among HAdV-F40 strains in Yantai was primarily attributed to point mutations rather than recombination events. This suggests that HAdV-F40 has remained genetically stable and relatively conserved in this region.

**Fig 4:**
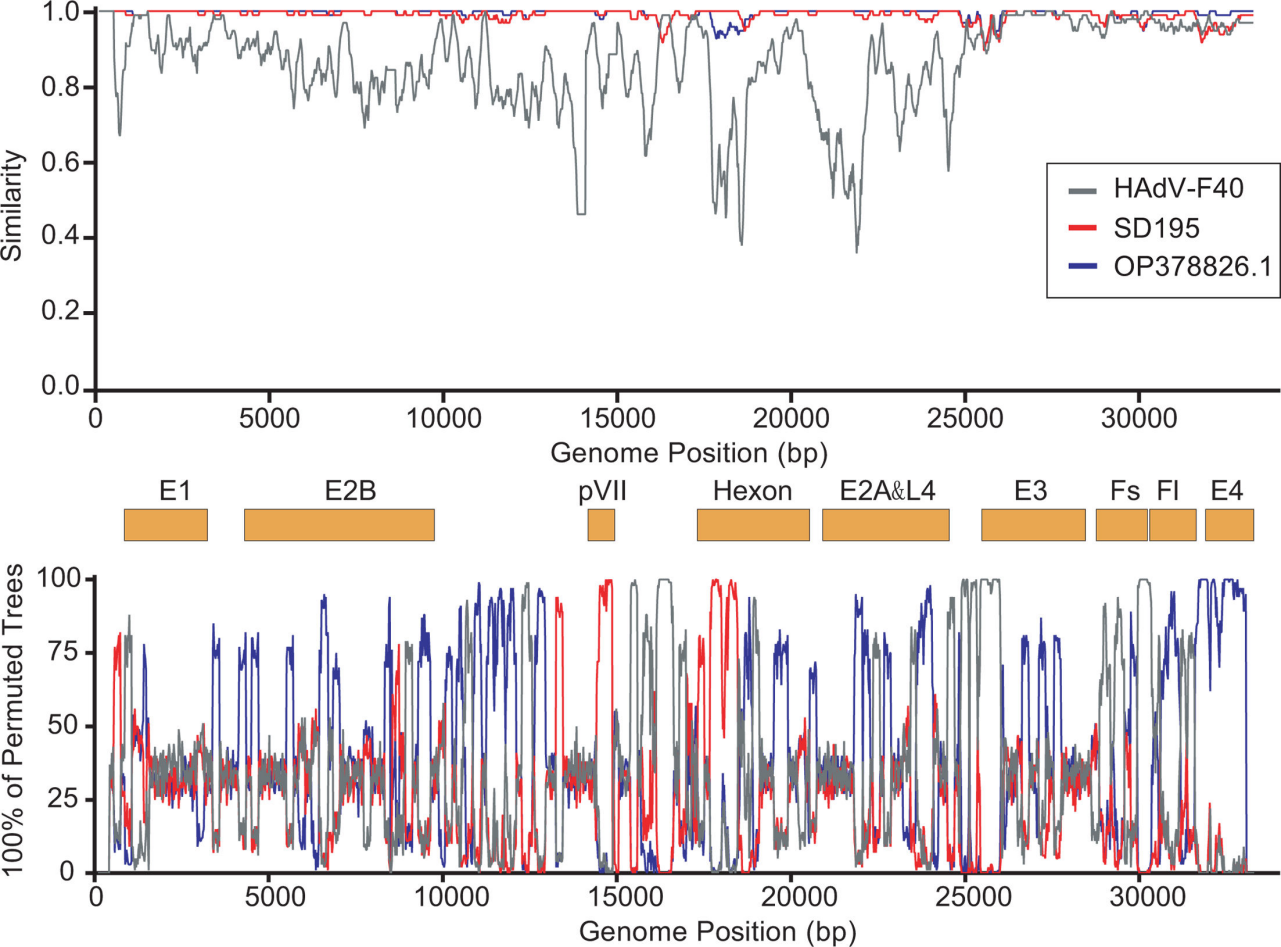
Recombination analysis of HAdV-F40/41 genomes. Recombination events in the HAdV-F40 and HAdV-F41 genomes were analyzed using SimPlot and Bootscan, with genomic positions plotted along the *x*-axis. The upper panel presents a similarity plot, showing sequence identity between SD195 and OP378826.1 relative to SD376, highlighting potential recombination breakpoints. The lower panel displays a Bootscan analysis, illustrating the percentage of permuted trees supporting recombination across various genomic regions, with peaks corresponding to recombination-prone loci. Key adenoviral genomic regions—including E1, E2B, pVII, hexon, E2A and L4, E3, Fs, Fl, and E4—are mapped along the *x*-axis, offering a comprehensive overview of potential recombination hotspots.

In contrast, HAdV-F41 displayed clear evidence of recombination, with one recombinant strain, SD376, identified as a product of genetic exchange between OP378826.1 (Major Parent) and SD195 (Minor Parent). The recombination breakpoints identified were predominantly located within the pVII and hexon genes, underscoring the occurrence of intra-subtype recombination within HAdV-F41 (Fig. 4).

Further lineage-based recombination analysis revealed inter-subtype recombination events between HAdV-F40 and HAdV-F41, with recombination breakpoints detected at 15,500–16,500 bp (L4 region), 24,500–26,000 bp (E3 region), and 30,000–30,500 bp (Fs region). These findings suggest that genetic exchange between HAdV-F40 and HAdV-F41 has played a significant role in genome diversification, highlighting the potential contribution of inter-subtype recombination to adenoviral evolution.

### Structural prediction and comparison of recombinant and reference strains

The 3D structure of the HAdV-F41 hexon protein was predicted using the AlphaFold3 platform, and the resulting model was compared to the existing cryo-EM structure (UniProtKB: P11820) to assess the accuracy of the AlphaFold3 predictions. Supplementary figure illustrates the comparison between the AlphaFold3-predicted structure (Fig. S1A) and the cryo-EM structure (Fig. S1B), revealing a high degree of structural similarity. This supports the reliability of the AlphaFold3 platform in generating accurate models for the hexon protein.

In light of the recombination analysis, which indicated that the recombination event in SD376 predominantly occurred at the pVII and hexon regions, we further investigated whether these specific regions exhibited structural changes compared to the reference strain OP378826.1. While the 3D structure prediction for hexon was successful, the prediction for pVII was inconclusive. The interface predicted template modeling score was <0.6, and the predicted template modeling score was <0.5 (11, 12), suggesting that reliable structural predictions for pVII could not be obtained.

**Fig 5:**
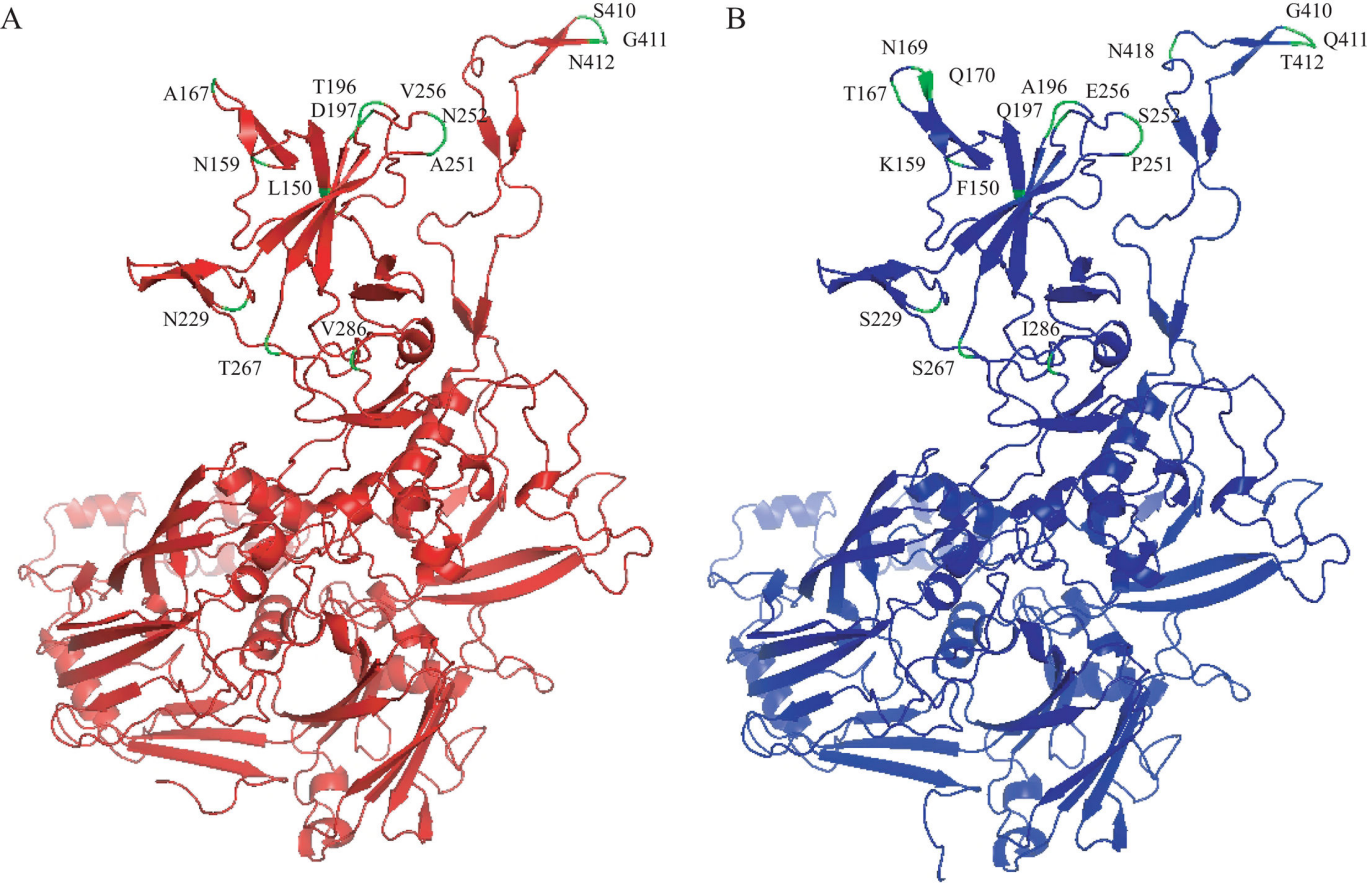
Structural comparison of hexon proteins from recombinant and reference strains. (**A**) 3D structure of the hexon protein from the recombinant strain SD376, displaying the overall spatial arrangement with key regions of interest highlighted. (**B**) 3D structure of the hexon protein from the reference strain OP378826.1, aligned for comparison with the recombinant strain SD376. The key differences in amino acid residues between the recombinant and reference strain hexon proteins are highlighted in green. A total of 14 mutations (L150, N159, A167, T196, D197, N229, A251, N252, V256, T267, V286, S410, G411, and N412) and three amino acid deletions (N169, Q170, and N418) were identified.

For the hexon protein, accurate 3D structure predictions were successfully generated, and structural modeling was conducted for both the recombinant strain SD376 and the reference strain OP378826.1. Figure 5 presents a structural comparison of the hexon proteins between the recombinant strain (Fig. 5A) and the reference strain (Fig. 5B). The comparison revealed no significant conformational changes between the two strains. However, 14 amino acid mutations (L150, N159, A167, T196, D197, N229, A251, N252, V256, T267, V286, S410, G411, and N412) and three amino acid deletions (N169, Q170, and N418) were identified, highlighting genetic variations between the recombinant and reference strains.

## DISCUSSION

In this study, we conducted a comprehensive molecular epidemiological investigation of HAdV-F40/41 circulating in Yantai, coastal Shandong, China (2017–2019). Our whole-genome sequencing revealed substantial genetic diversity, with local strains clustering into several distinct phylogenetic lineages. This indicates persistent co-circulation and ongoing evolution of HAdV-F40/41 in the region, similar to patterns seen in other Chinese cities such as Beijing and Hangzhou (3, 8).

Recombination serves as a crucial mechanism in adenovirus evolution, enabling the emergence of novel genotypes with altered pathogenicity (13). Our recombination analysis identified multiple putative breakpoints, particularly in the hexon and pVII regions, which may have implications for host cell tropism and immune evasion (14). These findings are consistent with reports from Brazil and Kenya, where recombination has been shown to drive the genetic diversification of circulating adenoviruses (2, 14). The emergence of recombinant strains, as observed in this and other studies, reinforces the need for continuous genomic monitoring (13).

Although our AlphaFold3 structural modeling indicated that the recombinant hexon protein did not undergo substantial conformational alterations, we identified several amino acid substitutions and deletions located within surface-exposed and hypervariable regions of the hexon. Previous studies have demonstrated that even subtle changes in these regions can significantly affect antibody recognition and immune escape (5, 10). For instance, substitutions at L150, N159, and N252, as well as deletions at N169 and Q170, may disrupt key neutralization epitopes, potentially facilitating persistent infection or evasion of pre-existing immunity. This is particularly relevant for pediatric populations, where herd immunity to adenovirus is variable. Although the precise functional consequences of these mutations remain to be elucidated, our findings underscore the importance of integrating genomic, immunological, and clinical data to fully understand the pathogenic and epidemiological significance of HAdV-F40/41 evolution. Continued genomic surveillance and further functional studies are warranted to clarify the clinical relevance of these mutations, especially with regard to vaccine design, immune escape, and disease burden in young children.

HAdV-F40/41 remains a major cause of AGE in children under five (1). High prevalence was observed in coastal Shandong during our study period, consistent with reports from Shanghai and Chongqing (4, 15). Cases increased in late autumn and winter, highlighting the need to better understand adenoviral seasonality (16). Clinically, HAdV-F40/41 causes diarrhea, vomiting, and dehydration, sometimes leading to severe complications (17). These findings underscore the importance of improved diagnostics and targeted interventions, such as vaccination and better sanitation, to reduce disease burden (9).

Several limitations should be considered in this study. First, this study was limited to a single city (Yantai), which may affect the generalizability of our findings to other regions. Second, sampling was restricted to symptomatic patients, excluding asymptomatic carriers and community cases, which could lead to underestimation of the true prevalence and diversity of HAdV-F40/41. Broader, multicenter studies including both symptomatic and asymptomatic individuals are needed for a more comprehensive understanding of HAdV-F40/41 epidemiology in China.

Given the significant genetic diversity and frequent recombination observed, ongoing genomic surveillance is essential to track viral evolution and potential changes in virulence or transmissibility. Integrating real-time sequencing with epidemiological data will enable early detection of new variants and inform timely public health responses (17). Although rotavirus vaccination has greatly reduced global gastroenteritis, adenovirus remains a major cause of pediatric diarrhea (7). Developing an effective HAdV-F40/41 vaccine will be crucial to further reduce disease burden (10).

## MATERIALS AND METHODS

### Collection and detection of adenovirus samples

Between January 2017 and December 2019, a total of 2,221 stool samples were systematically collected from patients diagnosed with AGE in Yantai, Shandong Province, China. These samples were obtained from hospitalized patients across a range of medical institutions. To ensure the specificity of the study, patients with confirmed bacterial or parasitic infections were excluded from the analysis. Ethical approval for this study was granted by the institutional review board, and informed consent was obtained from all participants or their legal guardians. Comprehensive clinical and demographic data were meticulously collected for each enrolled patient, including age, sex, residential area, and the date of sample collection. These data were recorded using a standardized case report form, thereby ensuring consistency, accuracy, and methodological rigor.

All stool samples were stored at −80°C until further processing. Detection of enteric adenoviruses was conducted using real-time fluorescence quantitative PCR (qPCR), targeting the conserved hexon gene. Samples exhibiting Ct values <40 were classified as positive for adenovirus. Rigorous quality control procedures, including the inclusion of both positive and negative controls, were implemented in each qPCR run to ensure the reliability and precision of the assay.

### Nucleic acid extraction and whole-genome sequencing

Viral DNA was extracted from stool samples using the QIAamp Viral DNA Mini Kit (Qiagen, Germany), in accordance with the manufacturer’s instructions. The concentration and purity of the extracted DNA were quantified using a Qubit Fluorometer (Thermo Fisher Scientific, USA), with only high-quality samples selected for subsequent whole-genome amplification and sequencing.

For the whole-genome amplification of HAdV-F40 and HAdV-F41, a commercial PCR amplification kit was employed to ensure comprehensive genome coverage. The resulting PCR products were purified using the QIAquick PCR Purification Kit (Qiagen, Germany) prior to sequencing.

Nanopore sequencing was performed following ligation-based library preparation, with sequencing carried out on the QNome-3841hex platform (Chengdu Qitan Technology Co., Ltd.). This process generated 18 Gb of sequencing data across six chips in 16 hours. For NGS, libraries were prepared using standard Illumina platform protocols, yielding high-throughput sequencing data.

The integration of nanopore and Illumina sequencing technologies enabled a thorough genomic characterization, facilitating detailed phylogenetic and recombination analyses.

### Genome assembly and annotation

To obtain complete genome sequences, nanopore sequencing reads were aligned to the reference genomes of HAdV-40 (GenBank accession: KU162869.1) and HAdV-41 (GenBank accession: AB728839.1) using Minimap2 (https://github.com/lh3/minimap2). Consensus sequences were generated with bcftools (https://github.com/samtools/bcftools/releases/), and mutation detection was performed using Medaka (https://github.com/nanoporetech/medaka). The resulting consensus sequences were meticulously refined based on the identified mutations to ensure high accuracy and reliability.

Sequence similarity to the reference genomes was evaluated using BLAST (version 2.12.0) to validate the genotype assignments. To further enhance accuracy, the assembled genomes were refined using NGS data with Pilon (https://github.com/broadinstitute/pilon), ensuring the generation of high-quality genome sequences. SNPs were identified using the iSNV-calling pipeline (https://github.com/generality/iSNVcalling), by aligning each NGS data set against the reference genome of the corresponding subtype.

### Phylogenetic and evolutionary analysis

To perform phylogenetic analysis and estimate divergence times, 30 HAdV-40 genomes and 133 HAdV-41 genomes were retrieved from the GenBank database for comparative analysis. Genomes of the same subtype were aligned with their respective reference sequences using BLAST (version 2.12.0), and multiple sequence alignments were generated using MView (version 1.67) (https://github.com/desmid/mview). A whole-genome phylogenetic tree was constructed employing the maximum likelihood (ML) method in IQ-TREE (https://github.com/iqtree/iqtree2), with robust bootstrap support to ensure the stability of the tree topology. Evolutionary history and divergence time were estimated using BEAST v1.8.2, implementing a single Markov chain Monte Carlo run of 10 million generations, with sampling every 200 generations. Temporal calibration was achieved by incorporating tip dates (terminal node calendar dates), and nucleotide substitution modeling was carried out using the General Time Reversible model with invariant sites and four gamma categories, applying default flat Dirichlet priors. An uncorrelated lognormal relaxed molecular clock, with a lognormal prior distribution, was utilized to estimate substitution rates. Post-analysis, a 10% burn-in was applied, and convergence was assessed using Tracer v1.7.1 to ensure that the effective sample size for all parameters exceeded 200. The final maximum clade credibility tree was generated with TreeAnnotator v1.8.2, calculating median node heights for divergence estimates. The resulting tree was visualized and annotated using FigTree v1.4.0.

### Recombination analysis

Recombination events in HAdV-F40 and HAdV-F41 were investigated using RDP5 and SimPlot, employing a comprehensive approach to detect and validate potential recombination signals. Full-genome alignments were initially imported into RDP5, where multiple recombination detection methods—including RDP, GENECONV, Bootscan, Maxchi, Chimera, and SiSscan—were applied. Analyses were conducted with a window size of 500 bp and a step size of 100 bp, ensuring high-resolution identification of potential recombination breakpoints. Only events with *P*-values < 0.05 were deemed statistically significant. To further validate and visualize the recombination signals, SimPlot was utilized to calculate sequence similarity using the Jukes-Cantor model. Bootscan plots were generated with a 500 bp window size and a 100 bp step size, providing graphical representation of sequence variation across suspected recombination regions.

### Structural modeling using AlphaFold3

The 3D structures of the recombinant strain and the reference strain were predicted using the AlphaFold3 platform. Structural differences between the recombinant and reference strains were analyzed to identify any variations resulting from the recombination event.

To assess the accuracy of the AlphaFold3 predictions, the 3D structure of the hexon protein from the previously published HAdV-F41 was also predicted using the same platform. The predicted structure was subsequently compared with the available cryo-EM structure (UniProtKB: P11820) to evaluate the precision of the model generated by AlphaFold3.

## Data Availability

The assembled genome sequences generated in this study have been deposited in GenBank under accession numbers PX453647-PX453680.

## SUPPLEMENTAL MATERIAL

Figure S1 aem.00983-25-s0001.pdf Structural comparison of HAdV-F41 hexon predicted by AlphaFold3 and cryo-EM.

Table S1 aem.00983-25-s0002.xlsx Distribution of human adenovirus subtypes detected in stool samples collected from patients with acute gastroenteritis in Yantai, Shandong Province, China.

ASM does not own the copyrights to Supplemental Material that may be linked to, or accessed through, an article. The authors have granted ASM a non-exclusive, world-wide license to publish the Supplemental Material files. Please contact the corresponding author directly for reuse.
