# Supplementary material for: Genetic diversity and phylogenetic characteristics of human adenovirus strains 40/41 circulating in Yantai, China, during 2017–2019
Source: Appl Environ Microbiol. 2025 Nov 25;91(12):e00983-25. doi: 10.1128/aem.00983-25 (PMC12724127; doi:10.1128/aem.00983-25)
Supplement: Figure S1 — Structural comparison of HAdV-F41 hexon predicted by AlphaFold3 and cryo-EM. [file aem.00983-25-s0001.pdf]

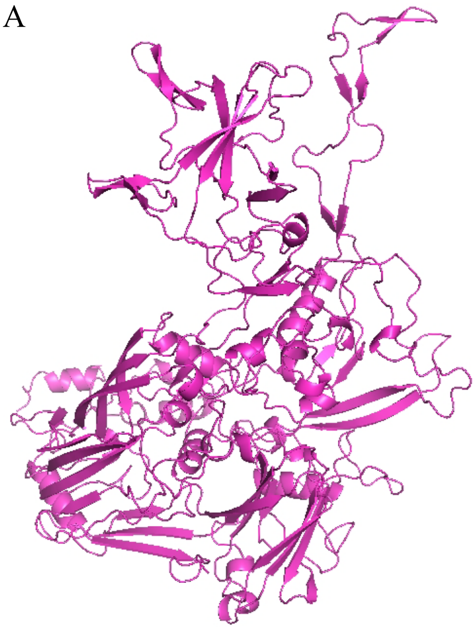

Supplementary Figure. Structural comparison of HAdV-F41 hexon predicted by AlphaFold3 and cryo-EM. (A) 3D structure of the hexon protein from HAdV-F41, predicted using the AlphaFold3 platform. The structural model is based on the published amino acid sequence of HAdV-F41, illustrating the overall spatial arrangement of the hexon protein. (B) 3D structure of the hexon protein from HAdV-F41, derived from the cryo-EM structure (UniProtKB: P11820). The cryo-EM structure is presented for comparison with the AlphaFold3-predicted model to evaluate the accuracy of the predicted structure.
